# Missing data in bioarchaeology II: A test of ordinal and continuous data imputation

**DOI:** 10.1002/ajpa.24614

**Published:** 2022-09-12

**Authors:** Amanda Wissler, Kelly E. Blevins, Jane E. Buikstra

**Affiliations:** ^1^ Department of Anthropology University of South Carolina Columbia South Carolina USA; ^2^ Archaeology Department Durham University Durham UK; ^3^ Center for Bioarchaeological Research, School of Human Evolution and Social Change Arizona State University Tempe Arizona USA

**Keywords:** bioarchaeology, imputation, missing data, paleopathology

## Abstract

**Objectives:**

Previous research has shown that while missing data are common in bioarchaeological studies, they are seldom handled using statistically rigorous methods. The primary objective of this article is to evaluate the ability of imputation to manage missing data and encourage the use of advanced statistical methods in bioarchaeology and paleopathology. An overview of missing data management in biological anthropology is provided, followed by a test of imputation and deletion methods for handling missing data.

**Materials and Methods:**

Missing data were simulated on complete datasets of ordinal (*n* = 287) and continuous (*n* = 369) bioarchaeological data. Missing values were imputed using five imputation methods (mean, predictive mean matching, random forest, expectation maximization, and stochastic regression) and the success of each at obtaining the parameters of the original dataset compared with pairwise and listwise deletion.

**Results:**

In all instances, listwise deletion was least successful at approximating the original parameters. Imputation of continuous data was more effective than ordinal data. Overall, no one method performed best and the amount of missing data proved a stronger predictor of imputation success.

**Discussion:**

These findings support the use of imputation methods over deletion for handling missing bioarchaeological and paleopathology data, especially when the data are continuous. Whereas deletion methods reduce sample size, imputation maintains sample size, improving statistical power and preventing bias from being introduced into the dataset.

## 1 INTRODUCTION

Missing data are ubiquitous in the social sciences. Bioarchaeological data may be lost due to myriad factors including differential preservation, selective excavation, post‐mortem damage, pathology, transcription errors, and/or computer crashes. When not handled properly, missing values can introduce substantial bias into a dataset, leading to erroneous study results and flawed interpretations (see Stojanowski & Johnson, 2015). Furthermore, most statistical tests require datasets with no missing data. Despite the significance of missing data, their treatment is often unreported in the social sciences, including in anthropology. When missing data are addressed, the least statistically and theoretically rigorous methods are generally used (see companion paper: Missing Data in Bioarchaeology I). The goal of this paper is to explore techniques for handling missing data, focusing on the use of imputation to manage missing bioarchaeological and paleopathological data. Imputation is defined as inserting a plausible value in place of a missing value (Allison, 2001; Schafer, 1999; Schafer & Graham, 2002). Our target audience includes anthropologists who have basic statistical and programming knowledge, but they need not be statistical experts, as methods are explained conceptually rather than mathematically. This paper has two sections: Part I gives a brief overview of classes of missing data and describes commonly used missing data methods in biological anthropology. Part II provides a case study test of seven methods for handling missing ordinal and continuous paleopathology data. This paper is intended as a companion paper to Missing Data in Bioarchaeology I, which reviews current approaches to missing data in bioarchaeology.

## 2 PART I: BACKGROUND

The best way to manage missing data depends on how and why the data are missing. Rubin (1976) described three main categories of missing data: missing completely at random, missing at random, and missing not at random. Data are described as missing completely at random (MCAR) when the reason the data are missing is unrelated to the pattern of missingness or any other variables of interest in the data set (Graham et al., 1997; Pepinsky, 2018; Quintero & LeBoulluec, 2018). If we have collected two variables (*X* and *Y*), data are MCAR “if the probability of missing data on *Y* is unrelated to the value of *Y* itself or to the values of any other variables in the data set” (Allison, 2001, p. 3). For example, in a dataset containing information on age (*X*) and femoral length (*Y*), data on femoral length would be MCAR if their missingness is unrelated to age or femoral length. MCAR data are likely rare among bioarchaeological datasets but could occur when skeletons are only partially recovered due to an incomplete excavation grid or when taphonomic processes vary stochastically across mortuary deposits, resulting in some poorly preserved skeletal elements or cortical surfaces.

The second category is missing at random (MAR). Data are missing at random if the pattern of missingness depends on some variable in the dataset that is not the variable of interest (Graham et al., 2007; Pepinsky, 2018; Quintero & LeBoulluec, 2018). Data are MAR if the probability of missing data on *Y* depends on the variable *X* but not on the value of *Y* (Allison, 2001). Using the above example, femoral length (*Y*) data would be missing at random if the missingness depended on age (*X*) but not on femoral length (*Y*) which could occur if older individuals more often had poorly preserved long bones due to osteoporosis.

The third category of missing data is missing not at random (MNAR), also called not missing at random (NMAR). Data are described as MNAR if the probability of missingness is related to the variable of interest, that is, if the probability of missing data on *Y* depends on *Y* itself (Pepinsky, 2018; Quintero & LeBoulluec, 2018). For example, data missing under the variable femoral length (*Y*) would be MNAR if the data are missing because of femoral length (*Y*). In practice, this may be because the researcher opted to exclude individuals with unusually short or long femurs, or because only “normal” femurs were accessioned into the collection (see Bhaskaran & Smeeth, 2014 for additional examples of MAR, MCAR, and MNAR variables).

Data that are MCAR or MAR are less problematic than MNAR and are often referred to as “ignorable” (Allison, 2001; Enders, 2010; Graham, 2012; Osborne, 2013). Deleting data that are MCAR or MAR, however, may result in a decline in statistical power due to a decreased sample size. Since MCAR and MAR data are distributed randomly, their absence should not introduce bias into the dataset (Graham, 2009; Howell, 2007; Myers, 2011). Data missing not at random, however, are problematic and referred to as “nonignorable” (Allison, 2001; Graham, 2009, 2012). The probability of missingness is dependent on the missing data, and it is almost impossible to know the true extent of that relationship. Therefore, it is not possible to control or compensate for data missing not at random (Graham, 2012; Howell, 2007; McKnight et al., 2007). MNAR data can result in a substantially biased dataset, because information vital to answering the research question is absent (De Leeuw et al., 2003; Finch, 2010; Graham, 2009; Osborne, 2013). In bioarchaeology (and paleopathology in particular), missing data likely fall into a combination of all three categories and it may be impossible to discern which variables belong in which category (Morris et al., 2014; Myers, 2011).

Overall, the methods bioarchaeologists use to manage missing data can broadly be classified into three categories: deletion, imputation, and maximum likelihood. Here we provide a detailed description of each approach, its advantages, and its disadvantages.

### 2.1 Deletion

1. Pairwise deletion (aka available case analysis) involves dropping cases or individuals based on variables present for each analysis (Allison, 2001; Graham, 2012; van Buuren, 2018). For example, an individual missing a periodontal disease score will be deleted from any analyses requiring periodontal disease as a variable but included in other tests, such as analyses for femoral length. This approach is easy to perform and has the benefit of making use of all available data, greatly maximizing the sample size. However, each analysis uses a slightly different sample, generating results that may not be comparable or are inconsistent across variables (Myers, 2011; Newman, 2014; van Ginkel et al., 2020). Published tables may list different sample sizes, which can be misleading if not appropriately explained, and repeatedly running similar analyses on overlapping samples raises concerns of alpha inflation. If the data are not MCAR, pairwise deletion can create bias in the parameter estimates (Allison, 2001; Baraldi & Enders, 2010). Furthermore, when each analysis is based on a slightly different sample, there is no straightforward procedure for calculating the standard error for the entire sample (Graham, 2012).
2. Listwise deletion (aka casewise deletion) involves the removal of an individual and all their data – an entire row in a spreadsheet – if any data for that individual are missing (Allison, 2001; Graham, 2012; van Buuren, 2018). This is the default method employed by statistical software programs SAS, SPSS, and Stata (van Buuren, 2018). Listwise deletion has the advantages of being easy to understand, being simple to execute, and not requiring advanced statistical knowledge or software (Allison, 2001; Meeyai, 2016). It creates a complete dataset that allows one to proceed with statistical analysis (Baraldi & Enders, 2010). If, however, the amount of missing data is even moderate, listwise deletion can result in an enormous decrease in sample size and subsequent loss of statistical power (Baraldi & Enders, 2010; Graham, 2012). The amount of missing data may be so great that entire variables could be deleted. If the data are not MCAR, listwise deletion can introduce substantial bias into final p‐values and confidence intervals (Allison, 2001; Baraldi & Enders, 2010). Many statisticians consider listwise deletion to be the worst of all possible techniques for handling missing data (Allison, 2001; King et al., 1998; van Buuren, 2018; Wilkinson, 1999).

### 2.2 Imputation

Imputation, defined as inserting a plausible value in place of a missing value, is an alternative to deletion methods for handling missing data (Allison, 2001; Schafer, 1999; Schafer & Graham, 2002). Imputation is a broad term that encompasses numerous frameworks and mathematical models for producing and selecting the imputed values. Critics of imputation claim these approaches “make up data,” invoking various justifications for continuing to use deletion methods (Osborne, 2013; Schafer, 1999; van Ginkel et al., 2020). Studies have shown, however, that imputed data are often better able to recover the original parameter estimates and are more easily replicable by other researchers than deletion methods (Fichman & Cummings, 2003; King et al., 1998; Osborne, 2013; Pedersen et al., 2017).

1. Mean replacement is the simplest imputation strategy: the mean of a variable is substituted for each missing data point of that variable (Little & Rubin, 2002). Mean imputation is easy to understand and implement but will decrease the variance of the sample. As such, large amounts of missing data will skew covariate relationships and affect the strength and direction of correlations (Graham, 2012; Musil et al., 2002; Osborne, 2013).
2. Regression imputation uses the complete case data for all variables to build a regression model that is then used to predict missing values. Regression imputation is conceptually intuitive and utilizes all variables in the dataset to generate predictions (Graham, 2012; Musil et al., 2002). The imputed values, however, lie on the regression line, so the sample variance is artificially decreased and correlations between variables are spuriously strengthened (Graham, 2012; van Buuren, 2018; Zhang, 2016).
3. Stochastic regression imputation corrects for the over correlation between variables by adding random “noise” back into the model (Newman, 2003; van Buuren, 2018). One way to add this noise is by randomly selecting from the residuals and adding that value to the predicted missing value (Enders, 2010; Little & Rubin, 2002; van Buuren, 2018). Stochastic regression has the advantage of being able to produce unbiased parameters when the data are MCAR or MAR but will underestimate standard errors (Allison, 2001; Enders, 2010).
4. Random forest (RF) imputation uses a decision tree approach to predict the best values to impute. A bootstrapped random subset of samples is created to build multiple regression trees for each variable (Shah et al., 2014). The behavior of the data as it is run through the trees predicts the best values for the missing data. RF imputation is a commonly used method in epidemiology (Henriksson et al., 2016; Shah et al., 2014; Weng et al., 2019) and is capable of handling mixed data types and variable interactions (Stekhoven & Bühlmann, 2012; Tang & Ishwaran, 2017; Waljee et al., 2013). RF imputation can also be perceived as a black box technique, with little understanding of how the decision trees are being grown (Breiman, 2001).
5. Predictive mean matching (PMM) is an expanded form of hot deck imputation. Hot deck imputation is a broad “record matching technique” in which missing values from an individual (the recipient) are replaced by observed values from a similar case (the donor) (Kaiser, 1983, p. 1). This method requires the selection of an imputation model, such as linear regression. The model is estimated using the complete cases of the predictor variable and the variable to be imputed, and then the model is used to predict all values of the variable to be imputed, observed, and missing. Subsequently, each predicted missing value is matched to the most similar predicted values from the observed cases; one of these close cases is randomly selected, and the missing value is substituted for the observed value (Bailey et al., 2020; Little, 1988; Vink et al., 2014). Because PMM matches values from other donor cases within the dataset, imputed values will always fit with the observed range of values (Kleinke, 2018; Vink et al., 2014). A potential disadvantage to PMM is that it may not be acceptable for use with small sample sizes, as the pool of available observed outcomes with a similar case prediction will be small (Kleinke, 2018).

### 2.3 Maximum likelihood estimation

1. Expectation maximization (EM) is a common maximum likelihood algorithm that uses a two‐step iterative process. In the E‐step, a missing value is imputed based on what would be expected given other values in the dataset (Dempster et al., 1977; Graham, 2012; Newman, 2003). In the M‐step, the algorithm checks whether the new value has the highest probability of being a good fit with the rest of data. If not, the process begins again, imputing a more likely value until all missing data have been replaced with the most likely values (Musil et al., 2002). EM procedures generally perform better than mean imputation or deletion methods (Nelwamondo et al., 2007). One potential drawback to EM, however, is that it produces *SE*s that may be narrower than those of the true data and thus may artificially increase statistical confidence (Musil et al., 2002).

### 2.4 Prior approaches to imputation in other disciplines

While missing data are a problem in nearly all fields of research, some disciplines, such as psychology, ecology, and health sciences, have adopted advanced methods for handling missing data more quickly than others, particularly compared to biological anthropology. We suspect this delay may be due to a lack of awareness that imputation exists or a belief that certain types of data (e.g., age and sex) are not appropriate to impute (McKnight et al., 2007). In other fields, however, scholars regularly impute a wide variety of missing demographic and social variables that are comparable to those used in biological anthropology and which can be used as a model for our field moving forward. Working in the social sciences, Evans and Smokowski (2015) tested how social capital—as measured by proxies such as social support and mental health—predicts the likelihood of intervening in school bullying. They imputed missing survey data on demographic factors such as ethnicity and religion, responses on parental support, school satisfaction, and optimism about the future. Turney (2015) examined how paternal incarceration may be a cause of food insecurity for children, imputing missing survey answers on how often a child has been hungry or how often they skipped meals.

Researchers in the natural and ecological sciences have adopted advanced techniques as standard for dealing with missing data, imputing a diverse array of biological traits—such as leaf area, seed mass, plant height, animal body mass, litter size, diet diversity, sociality, and generation length—that are analogous to data regularly used in many areas of biological anthropology (Bird et al., 2020; Cooke et al., 2019, 2020; Grilo et al., 2020; Ordonez & Svenning, 2017; Pacifici et al., 2013; Taugourdeau et al., 2014). Divíšek et al. (2018) for example, searched for patterns of traits that could be indicators of invasive plant species and imputed missing data on leaf area, plant height, and seed weight. While investigating how certain traits relate to ecological strategies, Cooke et al. (2020) used multiple imputation to manage missing data on body mass, habitat breadth, generation length, diet, and litter/clutch size.

Imputation of missing demographic or health data like those used in sociocultural anthropology and bioarchaeology is commonplace within epidemiological and clinical studies (Barnard & Meng, 1999; Bodnar et al., 2006; Costello et al., 2014; Ferrie et al., 2005; Petersen et al., 2014; Zeka et al., 2006). Lassale et al. (2018) imputed missing body measurements, testing the association between obesity and coronary heart disease. Dam et al. (2016) imputed missing values for age, education, smoking, and health status to examine whether increased alcohol use in postmenopausal women increases their risk of breast cancer while decreasing their risk of coronary heart disease. In general, disciplines in the social, ecological, and biological sciences employ more sophisticated approaches to missing data, particularly compared to certain areas in biological anthropology.

### 2.5 Prior approaches to imputation in biological anthropology

Missing data have been identified as a concern in many subfields of biological anthropology including paleoanthropology (Clavel et al., 2014; Gordon et al., 2008; Kramer & Konigsberg, 1999), primatology (Ely et al., 2013; Jardim et al., 2021), paleogenomics (Irving‐Pease et al., 2021; Ishiya et al., 2019; Mizuno et al., 2017), and bioarchaeology (Burnett et al., 2013; Kenyhercz & Passalacqua, 2016; Stojanowski & Johnson, 2015). While most scholars in these areas agree that deletion is an unsatisfactory method for handling missing data, there are few discipline‐specific papers providing guidance or suggesting best practices. As discussed in the companion paper, many researchers do not disclose the presence of missing values in their datasets. Imputation seems to have been adopted unequally among various subfields of biological anthropology, likely reflecting the types of questions asked, the statistical analyses employed, and the other disciplines from which each subfield draws.

Paleoanthropology relies on inherently fragmentary data and small sample sizes due to the nature of the fossil record. Paleoanthropologists have therefore been compelled to reconcile with their missing data more so than many other areas of biological anthropology. EM is a commonly found missing data method in vertebrate paleontology, having been recommended by Strauss et al. (2003) and used in numerous other studies in biological anthropology (Athreya & Wu, 2017; Scherer, 2007; Stefan, 2004). The need to reconstruct fragmentary hominin crania has been a major driver of missing data management. Glantz et al. (2009) imputed missing fossil cranial measurements to assess group membership for the Teshik‐Tash 1 cranium. Paleoanthropologists employing geometric morphometrics have adopted other approaches for handling missing values. Gunz et al. (2009) for example, proposed using multiple multivariate regression to estimate missing values to reconstruct fragmentary hominin crania.

Due to the fragmentary and low‐quantity nature of ancient DNA (aDNA), paleogeneticists must work with incomplete and low coverage genomic data, with most samples below an average 1x depth of coverage. Imputation is used to infer genotype calls (e.g., Aa, AA, or aa) across aDNA samples so that there are sufficient genotypes to compare among study samples and analyze concurrently with large comparative aDNA and modern datasets. Unlike other incomplete biological anthropology data, genomic data are helpfully governed by a well‐documented biological mechanism that allows genotypes to be inferred based on their association with other genotypes linkage disequilibrium (LD) (Neale, 2010). LD is the non‐random association alleles have with other geographically close alleles. Chromosomes recombine when chunks of the genome are exchanged during meiosis; these chunks are known as haplotypes. Therefore, genotype imputation methods leverage large reference panels of phased (separated by chromosome) haplotypes from hundreds to millions of individuals to infer missing genotypes (Browning & Browning, 2016). While genotype imputation is highly successful for modern genomic data (Browning et al., 2018; Pasaniuc et al., 2012), there are caveats when working with aDNA. Namely, high‐quality comparative reference panels comprise genomes from modern individuals, which may not be representative of ancient genomic diversity and consequently bias results toward the reference alleles (Hui et al., 2020). Also, miscoding lesions sequenced from degraded aDNA fragments and sequencing errors in low coverage aDNA data can masquerade as variants, which can penalize real genotype similarities and add additional noise to sparse and stochastically preserved aDNA data. Despite these difficulties, researchers have used imputation workflows to significantly increase aDNA sample sizes for downstream analyses, such as population affinity, genetic relatedness, demographic history, and phenotypic inferences (Gamba et al., 2014; Jensen et al., 2019; Martiniano et al., 2017).

Primatologists have also dealt with missing data in their research, though the qualities of those data and causes of their missingness often differ from those in paleogenomics or paleoanthropology. While investigating the social pairing process among rhesus macaques Capitanio et al. (2017) imputed missing values on behavioral responsiveness and temperament. Grebe et al. (2019) studied ringtail lemurs to understand mechanisms and origins of dominance among females. Using the AMELIA package in R, the authors not only imputed missing endocrine data, but also compared imputed and observed values to ensure they had generated plausible imputed values. Studying the sleeping behaviors of proboscis monkeys, Feilen and Marshall (2014) imputed missing values on tree characteristics and measurements. The authors additionally noted the reasons for missing data that included “malfunction of data collection devices, forest fires, river closure, and storms” (p. 1132) which hindered data collection.

Bioarchaeological data have their own suite of unique characteristics that can make them more challenging to analyze than data from other fields. The data are often a mix of continuous, categorical, and binary variables that are best analyzed together. Many statistical tests do not work well with categorical data or do not accept mixed data types. Unlike continuous data, categorical and binary data have a low range of possible values. For example, according to “Standards for Data Collection from Human Skeletal Remains” (Buikstra & Ubelaker, 1994), porotic hyperostosis should be recorded as 0, 1, 2, 3, or 4 – with 0 as no expression, and 4 as the highest expression. Some of the more statistically complicated methods for imputing missing data do not work well with such a narrow range of allowable values. However, because of this low range, less computationally intensive methods may be successful; for instance, a randomly imputed number selected from 0 to 4 is more likely to fit than one selected from 0 to 100. Furthermore, the missing values in a bioarchaeological dataset may fall in different classes of missingness depending on the variable. Cribra orbitalia may be MCAR, linear enamel hypoplasia may be MAR, and periodontal disease may be MNAR. Each variable may require separate pre‐analysis data treatments and procedures for handling missing values (Stojanowski & Johnson, 2015).

Another challenge with bioarchaeological data is that we regularly collect data that are MNAR, yet we fail to account for those biases in our analyses or interpretations. For example, most scoring procedures for periodontal disease code missing teeth as NA or not scorable (e.g., Kerr, 1988). However, in cases of extreme periodontal disease, tooth loss will occur (Lindhe et al., 1983; Morelli et al., 2018; Ong, 1998; Ramseier et al., 2017). Antemortem tooth loss may therefore be the highest expression of periodontal disease. Scoring teeth missing antemortem as NA introduces MNAR values, creating a biased dataset.

Compared to other subtopics within biological anthropology, bioarchaeologists have made far less use of statistically sophisticated methods for handling missing data and are more likely to rely on deletion methods (see companion article). The areas in which imputation has been used extensively include biodistance analyses and broader investigations of population affinity (Godde & Rangel González, 2022; Paul et al., 2013; Prevedorou & Stojanowski, 2017; Rathmann et al., 2022). Noting the limitations of missing data early on, Howells (1973) proposed three options for handling missing biodistance data: mean imputation, regression, and making an educated guess. Working with dental metrics and nonmetrics, Thompson et al. (2015) imputed missing values to reevaluate evidence surrounding biological relatedness and population movement at Cahokia's mound 72. Similarly, Redfern and Hefner (2019) imputed missing cranial measurements to investigate the presence of individuals with African ancestry in the East Smithfield Black Death cemetery. In recent years, numerous dissertations have emerged that impute missing biodistance data (Bethard, 2013; Bolhofner, 2017; Miller, 2015; Pacheco‐Forés, 2020; Paul, 2017).

Despite the regular use of imputation in biodistance, few researchers have assessed the performance of imputed data when analyzed. Kenyhercz and Passalacqua (2016) tested four different imputation methods—hot deck, iterative robust model‐based imputation (IMRI), *k*‐nearest neighbor (kNN), and mean—on continuous cranial metric data with 10%, 20%, 50%, and 90% of the values in the dataset missing. Kenyhercza et al. (2019) performed a nearly identical study but assessing imputation of ordinal nonmetric cranial traits. Both papers found all imputation methods perform similarly with low amounts of missing data. At higher percentages of missing data, however, differences emerged; kNN performed well with cranial metric data and IRMI worked well with cranial nonmetric data. Kenyhercz and Passalacqua (2016) however, additionally tested how imputed data affect biodistance analyses by calculating Mahalanobis distances (*D* ^2^) using both imputed and complete datasets. They found that hot deck, IRMI, and mean imputation artificially decreased the distance between populations, causing them to appear more similar while kNN increased the distance. Fortunately, most methods generated values that were able to classify individuals into correct population groupings with acceptable success. IRMI, however, had the highest levels of group misclassification, with one group only receiving a 7% correct classification.

Beyond biodistance, few researchers have investigated imputation of other bioarchaeological data such as pathology, trauma, age‐at‐death, or sex. Auerbach and colleagues (Auerbach, 2011; Auerbach et al., 2005; Auerbach & Ruff, 2004) proposed several multiple regression equations to estimate missing skeletal measurements to assess stature. Wissler (2021) imputed missing ordinal paleopathology data to investigate frailty and survival in the 1918 influenza pandemic. A recent paper by Muzzall (2021) proposed a technique for estimating biological sex. Using machine learning techniques and generalized low rank models to impute missing data, the model achieved a high success rate when cranial interlandmarks and dental metric distances are combined.

## 3 PART II: A CASE STUDY TEST OF IMPUTATION OF PALEOPATHOLOGY DATA

The second aim of this paper is to discover which imputation techniques are appropriate for imputing missing ordinal and continuous paleopathology data. Previous researchers have noted that the amount of missing data can have substantial impacts on representativeness of a dataset as well as the success of various imputation approaches (Kleinke, 2018; Leite & Beretvas, 2010; Quintero & LeBoulluec, 2018). Furthermore, whether the data are MCAR, MAR, or MNAR will affect imputation performance (King et al., 1998; Musil et al., 2002; Pepinsky, 2018). We therefore examine how different amounts of missing data and how patterns of missingness impact imputation success. To accomplish this, we simulated missing data on two complete bioarchaeological datasets (no missing data) and tested five methods for imputing ordinal paleopathology data and continuous skeletal measurements alongside pairwise and listwise deletion to discover which approach best approximated the parameters of the original dataset.

### 3.1 Materials

Ordinal missing data were simulated on a complete dataset of 287 individuals from the Hamann–Todd Human Skeletal Collection. This sample includes a mix of males, females, African American individuals, and European American individuals ranging in age from 18 to 80 years. Recorded paleopathology data include porotic hyperostosis, cribra orbitalia, periodontal disease, linear enamel hypoplasia, and periosteal lesions of the tibia. The range of ordinal values for each are porotic hyperostosis, 0–2; cribra orbitalia, 0–3; periodontal disease, 0–4; linear enamel hypoplasia, 0–3; and periostosis, 0–3.

Continuous missing data were simulated on a complete dataset of 369 individuals from the same collection. Variables include left and right femoral bicondylar lengths – measured in centimeters – and the antero‐posterior (AP) and transverse (TR) vertebral neural canal diameters of the first, fifth, and tenth thoracic vertebrae (T1, T5, and T10), and the first and third lumbar vertebrae (L1 and L3) – measured in millimeters.

### 3.2 Methods

Seven missing data methods were chosen for evaluation: mean imputation, PMM, stochastic linear regression, RF, EM, pairwise deletion, and listwise deletion. These methods were chosen because they are commonly used in the social sciences. Excellent statistical packages are available for each, making these methods easy to implement and more accessible to non‐experts. These methods represent a wide range of statistical approaches and range from mathematically simple (e.g., mean imputation), to complex (e.g., EM).

Several R packages were used to impute the missing data, as no single package worked with all methods and all data types. Mean replacement, PMM, and stochastic linear regression were achieved using the mice package (v3.11.0; van Buuren & Groothuis‐Oudshoorn, 2011). For each, *m* = 10 imputations were performed with 50 iterations. RF imputation was executed with the missForest package (v1.4; Stekhoven & Bühlmann, 2012) for both ordinal and continuous data; the mice package was unable to form discrete decision trees for the ordinal data given the low range of possible values. EM for both ordinal and continuous data was performed using missMethods (v0.2.0; Rockel, 2020). Pairwise deletion was achieved using na.rm = TRUE to remove individuals with missing values by variable. Listwise deletion used the na.omit function, deleting an entire individual from that iteration of the dataset. All analyses were performed in RStudio version 1.1.456 (Rstudio Team, 2016).

To evaluate how the amount of missing data influences the success of the seven approaches five different datasets with 5%, 10%, 20%, 30%, and 40% of the data missing were created using the R package imputeR (v2.2; Feng et al., 2020) resulting in 25 ordinal datasets and 25 continuous datasets with missing data. To assess how patterns in the missing data affect imputation, five additional datasets with percentages of missingness that differ for each variable were created to more accurately reflect patterns of missingness found in a genuine bioarchaeology dataset. Missing data were simulated as MCAR, MAR, and MNAR using the R package missMethods (v0.2.0; Rockel, 2020), resulting in a total of 15 additional datasets. For the ordinal data, percentages of missingness were set at porotic hyperostosis = 12.5%, cribra orbitalia = 20%, periodontal disease = 25%, linear enamel hypoplasia = 30%, and periostosis = 10%. For continuous data, the following percentages of missingness were selected: femoral length right = 10%, femoral length left = 10%, T1AP = 15%, T1TR = 10%, T5AP = 12.5%, T5TR = 10%, T10AP = 15%, T10TR = 15%, L1AP = 20%, L1TR = 15%, L3AP = 20%, and L3TR = 15%. These percentages mirror the amount of missing values in the first author's dissertation dataset (Wissler, 2021). Note that these data come from a documented osteological collection and therefore these percentages may not represent what would be found in an archeological assemblage.

### 3.3 Assessing success

The success of each imputation method was assessed using the normalized root mean square error (NRMSE), which measures the difference between predicted and observed values; a lower NRMSE indicates a better fit. NRMSE was calculated using the hydroGOF package (v0.4‐0; Zambrano‐Bigiarini, 2020). As NRMSE is a standard metric for evaluating imputation methods, the results will be broadly comparable to similar studies in other disciplines. Calculating NRMSE requires original and imputed values and thus could not be used to assess success for the two deletion methods. Therefore, percent error of the mean was also used to compare success of imputation and deletion methods. NRMSE and percent error quantify slightly different aspects of imputation success. NRMSE quantifies the difference between paired original and imputed values; percent error evaluates the difference in the overall mean between the original and imputed datasets. The R code for these procedures is available under the first author's GitHub Repository (Wissler, 2022).

### 3.4 Case study results

**FIGURE 1:**
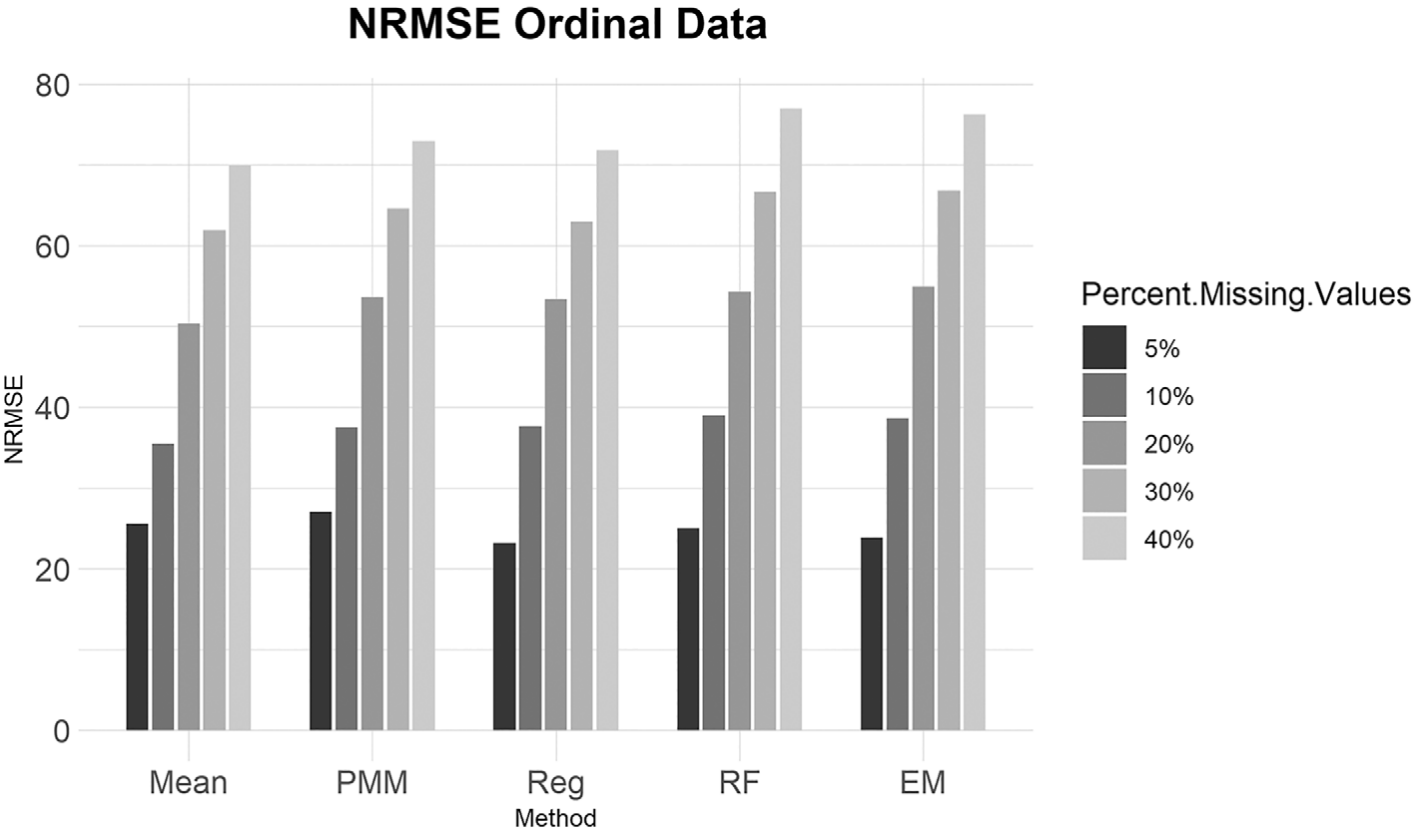
Barplot showing imputation results using normalized root mean square error (NRMSE) for ordinal data with percent missing values. EM, expectation maximization; Mean, mean; PMM, predictive mean matching; Reg, regression; RF, random forest

**FIGURE 2:**
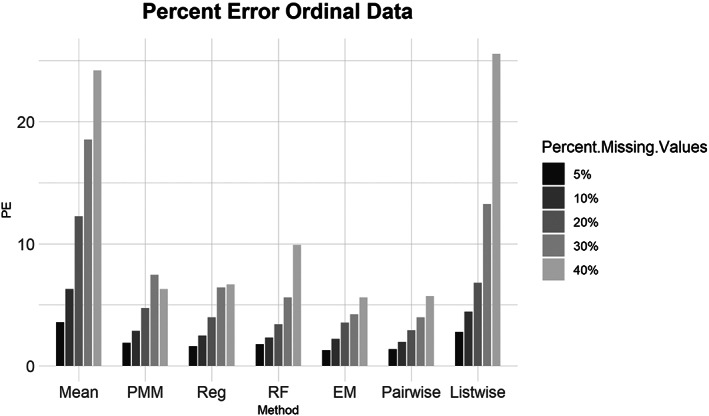
Barplot showing imputation results using percent error for ordinal data with percent missing values. EM, expectation maximization; Listwise, listwise deletion; Mean, mean; pairwise, pairwise deletion; PMM, predictive mean matching; Reg, regression; RF, random forest

**FIGURE 3:**
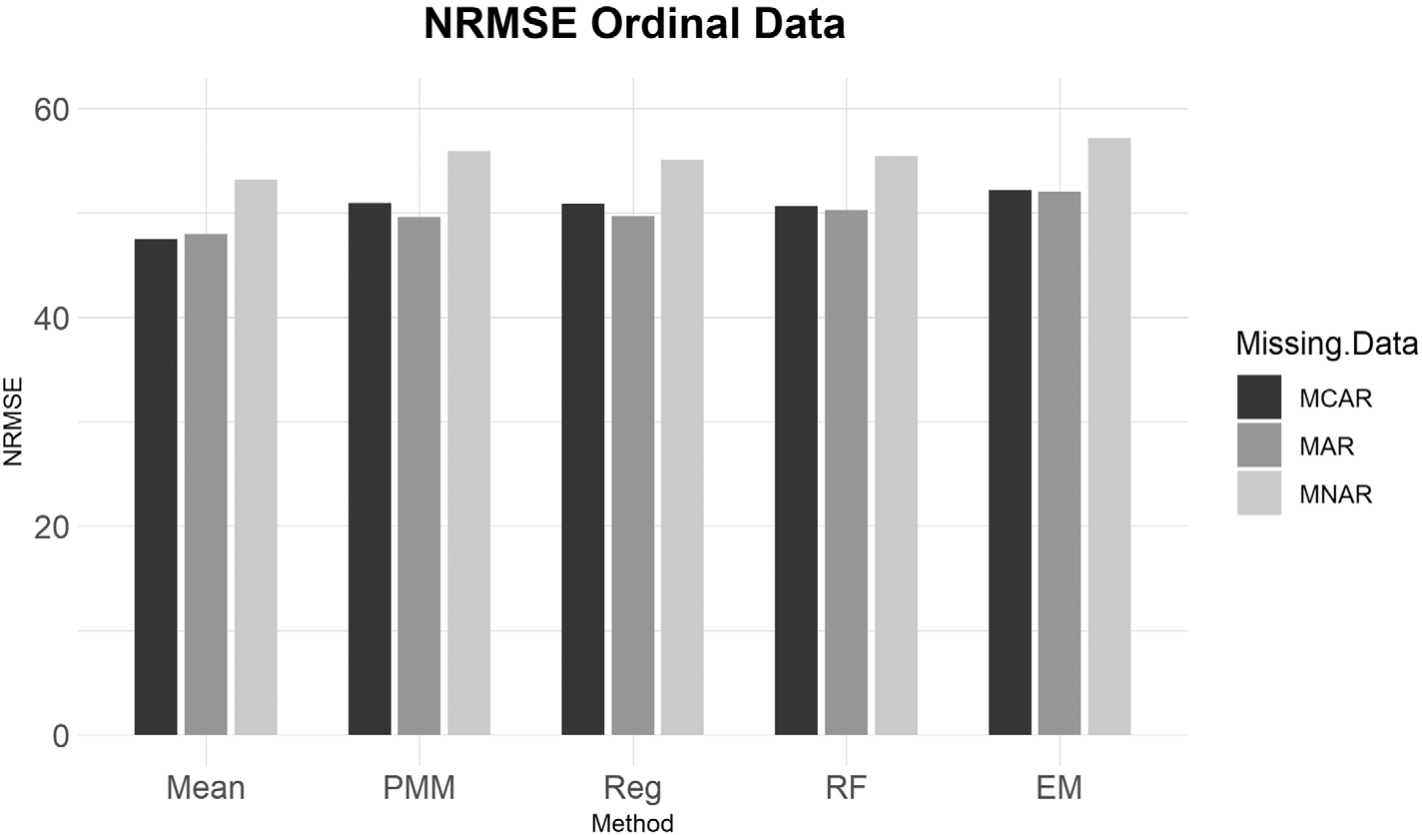
Barplot showing imputation results using normalized root mean square error (NRMSE) for ordinal data with missing completely at random (MCAR), missing at random (MAR), and missing not at random (MNAR) missing data. EM, expectation maximization; Mean, mean; PMM, predictive mean matching; Reg, regression; RF, random forest

**FIGURE 4:**
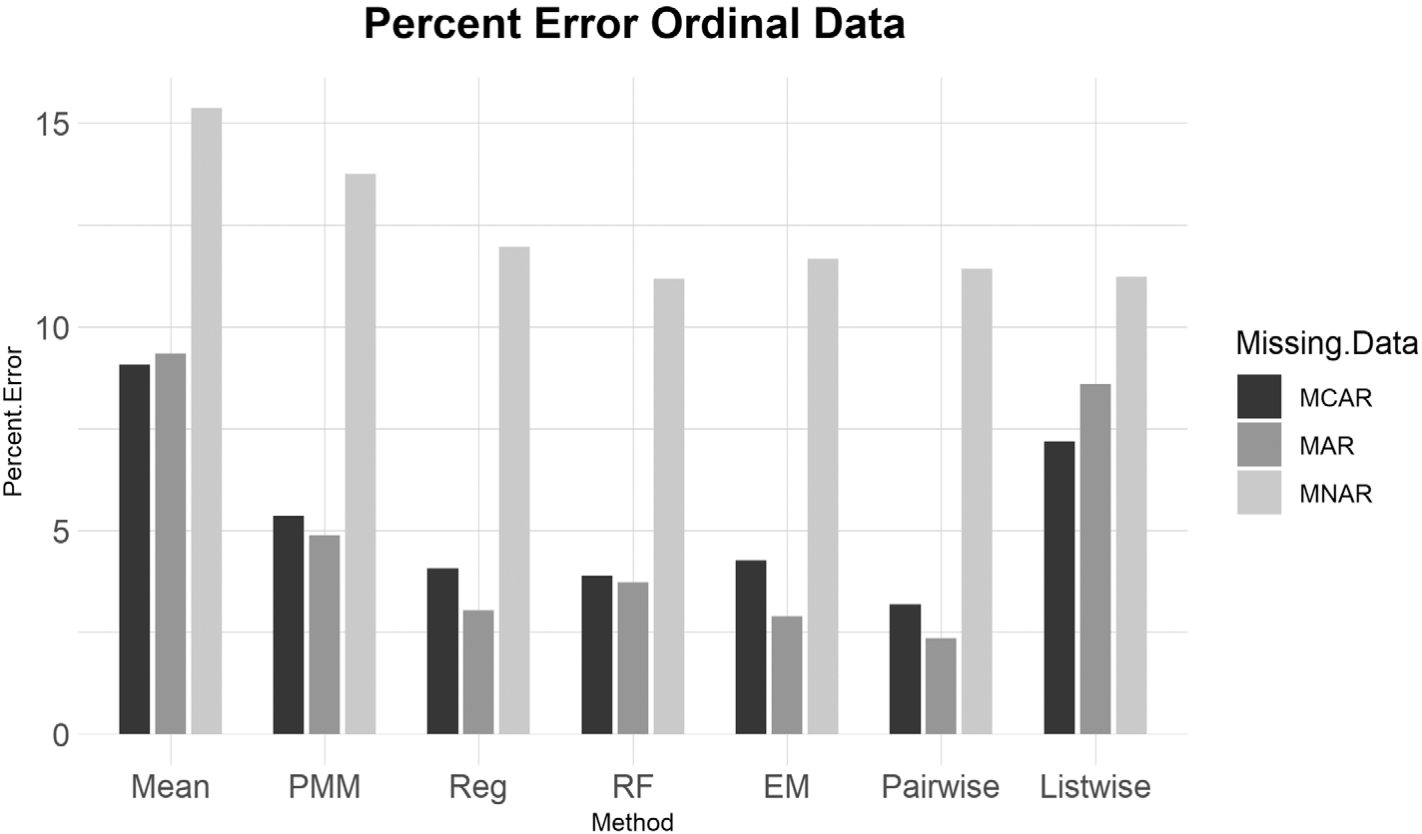
Barplot showing imputation results using percent error for ordinal data with missing completely at random (MCAR), missing at random (MAR), and missing not at random (MNAR) missing data. EM, expectation maximization; Listwise, listwise deletion; Mean, mean; Pairwise, pairwise deletion; PMM, predictive mean matching; Reg, regression; RF, random forest

1. Ordinal data summary results are shown in Figures 1, 2, 3, 4. Tables with the complete results are available as Supporting Information. Figure 1 shows that when evaluated using NRMSE, all imputation methods performed roughly the same. For the 5%, 10%, 20%, 30%, and 40% missing datasets, pairwise deletion, and EM had slightly better performance compared to the other methods while listwise deletion was the worst when evaluated with percent error (Figure 2). Mean imputation of ordinal data performed poorly compared to all other imputation and deletion methods at 5%, 10%, 20%, and 30% missingness when evaluated with percent error. PMM with 30% missingness did worse than 40% missingness when assessed with percent error, which is not a pattern that is found among any other results.

Similarly, for the MCAR, MAR, and MNAR datasets, all methods performed about the same based on the NRMSE (Figure 3). Interestingly, there is no strong difference in imputation success among data that are MCAR, MAR, and MNAR under NRMSE, which contrasts with the findings of other similar studies (Musil et al., 2002; Pepinsky, 2018). Evaluated with percent error, however, all the missing data methods perform worse on MNAR datasets compared to MCAR or MAR datasets (Figure 4). Overall, no single imputation method was best able to recover the parameters of the original dataset in all categories of missing ordinal data.

**FIGURE 5:**
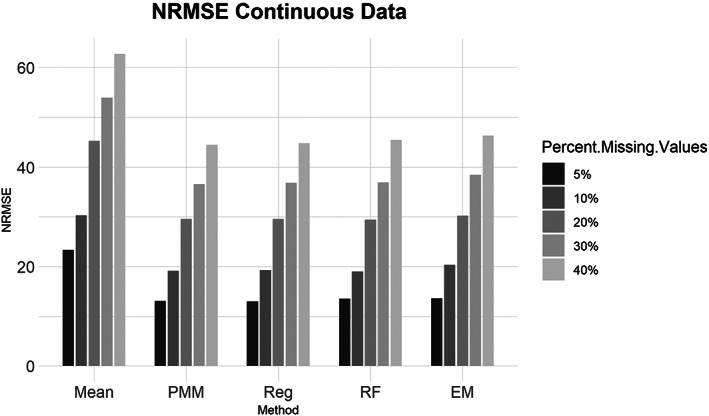
Barplot showing imputation results using normalized root mean square error (NRMSE) for continuous data with percent missing values. EM, expectation maximization; Mean, mean; PMM, predictive mean matching; Reg, regression; RF, random forest

**FIGURE 6:**
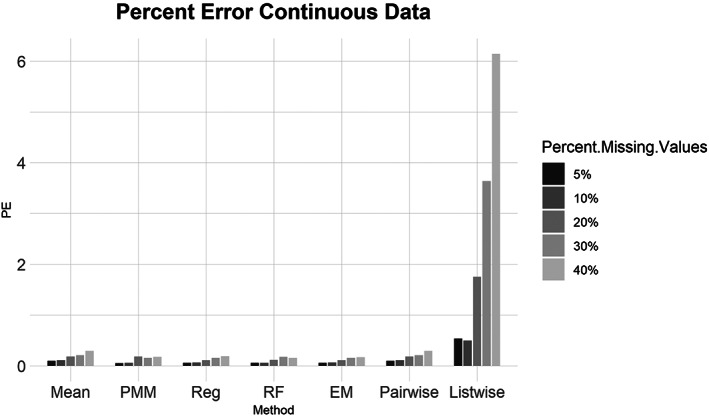
Barplot showing imputation results using percent error for continuous data with percent missing values. EM, expectation maximization; Listwise, listwise deletion; Mean, mean; Pairwise, pairwise deletion; PMM, predictive mean matching; Reg, regression; RF, random forest

**FIGURE 7:**
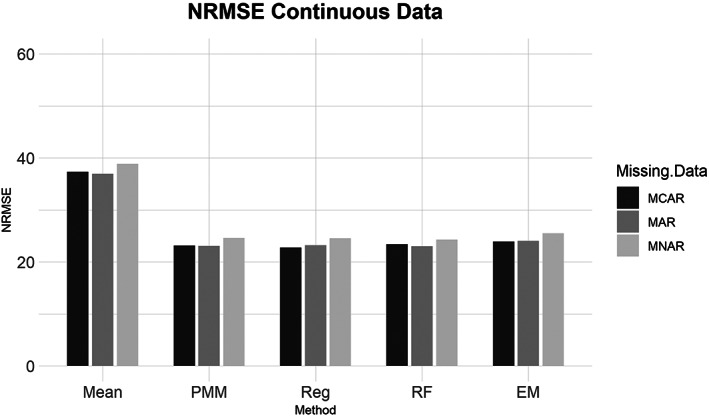
Barplot showing imputation results using normalized root mean square error (NRMSE) for continuous data with missing completely at random (MCAR), missing at random (MAR), and missing not at random (MNAR) missing data. EM, expectation maximization; Mean, mean; PMM, predictive mean matching; Reg, regression; RF, random forest

**FIGURE 8:**
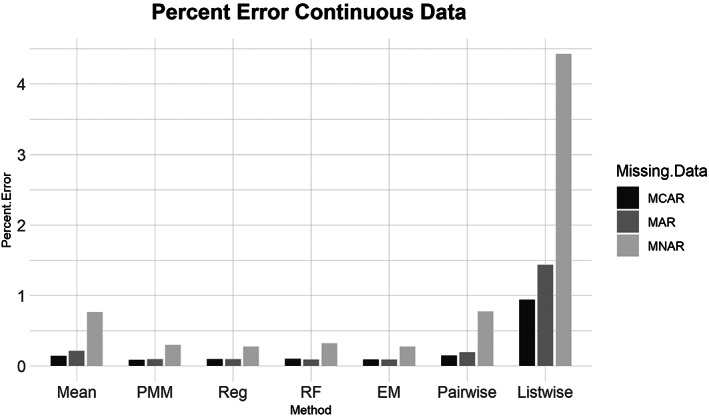
Barplot showing imputation results using percent error for continuous data with missing completely at random (MCAR), missing at random (MAR), and missing not at random (MNAR) missing data. EM, expectation maximization; Listwise, listwise deletion; Mean, mean; Pairwise, pairwise deletion; PMM, predictive mean matching; Reg, regression; RF, random forest

2 Continuous data summary results are shown in Figures 5, 6, 7, 8. Tables with the complete results are available as Supporting Information. The results for continuous data are similar to those of the ordinal data. For 5%, 10%, 20%, 30%, and 40% missingness, the percent of missing data was a stronger predictor of imputation success than the imputation method, with the possible exception of mean imputation, which performed worse than all other methods across all levels of missingness (Figure 5). Listwise deletion performed considerably worse compared to all other methods; the percent error for even 5% missing data with listwise deletion exceeded the percent error for 40% missingness with any other method (Figure 6). Overall, data that are MNAR generally have worse imputation success regardless of the method, though the differences are not large when assessed using NRMSE (Figure 7). Figure 8 likewise shows that listwise deletion has the least success at obtaining the parameters of the original dataset, as even MCAR data had a higher percent error when treated with listwise deletion compared to MNAR data with any other missing data method.
3 General findings across all the results – ordinal and continuous – there is little variation in performance apart from mean imputation and listwise deletion. When evaluated with NRMSE, mean imputation for continuous data performed worse across all amounts of missingness (5%–40%) and all patterns of missingness (MCAR, MAR, and MNAR) (Figures 5 and 7). With ordinal data, however, mean imputation was among the better performing methods (Figures 1 and 3), although the difference is not great. Using percent error, however, mean imputation is among the best‐performing methods, except for data that are MNAR. This discrepancy is due to how NRMSE and percent error are calculated. NRMSE assesses differences between paired original and imputed values while percent error assesses differences in the overall mean between the original and imputed datasets. As most of the imputation methods used here do not calculate imputed values using the mean (e.g., random forest, stochastic regression, and EM), percent error may produce results that are slightly biased against these approaches. Whether NRMSE or percent error is better for evaluating the success of missing data methods will depend on whether one is trying to obtain the exact values of the original dataset or retain overall patterns in the data.

Listwise deletion was undoubtedly the worst method for handling missing data. The percent error for even 5% or 10% missing data exceeded that of 40% missingness for the six other methods. Note that for continuous data, listwise deletion with 40% missingness had a percent error of only 6.14% (exact percent errors are available in Supporting Information). With ordinal data, even the more sophisticated methods had percent errors between 3.9 and 7.4 for 30% missingness. Furthermore, once listwise deletion had been performed with 40% of the values missing there were only a handful of individuals (rows) left in the dataframes, and in two versions the entire dataframe was empty as all rows had at least one NA.

On the whole, all imputation methods were relatively successful at recovering the means of the MCAR and MAR datasets. More sophisticated forms of imputation (PMM, regression, random forest, and EM) performed much better than mean imputation or either deletion method.

## 4 DISCUSSION

For both continuous and ordinal data, no imputation or deletion method performed noticeably better than any others across all datasets. Overall, evaluating success of ordinal data proved more difficult than continuous data; the results are more inconsistent and even minor differences in which values were simulated as missing—thus affecting the underlying distribution of the datasets—seemed to have a greater impact on the final results. The success of all seven missing data methods was much worse for ordinal data. Even the lowest percent error for 5% missing ordinal data was greater than the percent error for 40% missing continuous data. This likely reflects problems inherent in ordinal paleopathology data: the low range of possible values and variable percentages of missingness. It is possible that ordinal paleopathology data are not well‐suited for imputation. Imputation assumes that there are associations among variables in a dataset. If those associations are poor or absent, imputation will not work. Liao et al. (2014) have devised an “imputability measure” that “provides quantitative evidence of how well each missing value can be imputed by borrowing information from other variables or subjects” (p. 355). The authors warn that researchers should be cautious with variables that have poor imputability measures. While beyond the scope of this paper, it would be worth investigating the performance of ordinal paleopathology data when evaluated with this imputability measure.

An additional concern with the imputation of ordinal data is when scores are collapsed to binary variables such as presence/absence. There is little guidance on whether dichotomization should occur before imputation or after (Demirtas, 2007; Floden & Bell, 2019). Grobler and Lee (2020) found that imputing the original value and dichotomizing at the end can result in biased parameter estimates. They recommend “imput[ing] the binary variable even if intuitively this means throwing away potentially useful data” (p. 476). Furthermore, depending on the method used, imputed values of ordinal variables may be converted to continuous variables which do not match the values required for analysis. As an example, if the values of an ordinal dataset range from 0 to 2, imputed values may be 1.2 or 0.7, neither of which are allowable values. In such circumstances, practitioners often round to the nearest whole integer that matches the data (Schafer, 1997). Horton et al. (2003) however, found that rounding may lead to inaccurate parameter estimates if the data are not normally distributed. Simple rounding is highly discouraged for nominal datasets because it imposes an order on the data that is not present in the original dataset (Galati et al., 2014).

It has been noted that some of the “ordinal” data bioarchaeologists collect may actually be nominal, that is, scores thought to have inherent ranking may not be so organized. Hens and Godde (2020) found that increasing palatal suture closure scores did not consistently reflect greater age at death and were therefore nominal rather than ordinal scores. Imputation of nominal data has received considerably less attention than ordinal data (Lang & Wu, 2017). As mentioned above, imputing non‐ordinal variables may impose an order on the dataset that should not be present. Additional research is needed to clarify how imputation of nominal data impacts the dataset and parameter estimates.

Despite expert caution against pairwise deletion (Allison, 2001; Graham, 2012; Kang, 2013; van Buuren, 2018), this method performed reasonably well for both ordinal and continuous data, though it is difficult to fully test its success as it could not be assessed with NRMSE. Deletion methods cause a high rate of data loss that can be especially problematic if the data are MNAR. As paleopathologists generally have small sample sizes, any method that reduces the data further is suboptimal and may result in a biased dataset and decreased analytical power. The findings of this study agree with prior research on the use of listwise deletion (e.g., King et al., 1998). Listwise deletion was the worst at recovering the parameters of the original skeletal sample, particularly if the data are MNAR.

How much missing data is too much? There is little clear guidance on the maximum amount of missing data allowed before missing data methods or statistical analyses become too biased (Dong & Peng, 2013; Hardt et al., 2013; Meeyai, 2016; Saunders et al., 2006). The definition of a “small” amount of missingness varies from <5% to <20% missing (Little & Rubin, 2002; Tabachnick et al., 2007). Some statisticians caution that bias may occur in samples with more than 10% of the data missing and that samples with over 40% missing should be used for “hypothesis generating” only (Madley‐Dowd et al., 2019, p. 64). Others recommend a maximum of 30% missing when imputing missing values and no more than 20% with sample sizes of 50 or lower (Hardt et al., 2013). Under tightly controlled circumstances, however, authors have managed to successfully impute and analyze datasets with much higher percentages of missing data. Madley‐Dowd et al. (2019) imputed up to 80% missing MCAR and MAR data employing multiple imputation with auxiliary variables. Meeyai (2016) was able to recover unbiased parameters for MCAR data with 60% of the values missing for samples greater than *n* = 1000. Unbiased regression coefficients have been obtained with a 90% fraction of missing information,^1^ though statistical power dropped considerably after 50% missing even with *m* = 20 imputations (Graham et al., 2007).

The percent of missing information may not be the most important consideration when faced with missing data. Sample size is an important factor; 20% missing may have a greater impact with a sample size of 50 than with a sample size of 500 (Meeyai, 2016; Saunders et al., 2006). The class of missingness – MCAR, MAR, or MNAR – will also affect how much missing data is acceptable. Even a small amount of MNAR values may result in a biased dataset no matter what imputation method is used (Dong & Peng, 2013; Tabachnick et al., 2007). Whether the missing values are among the independent and/or dependent variables will also impact the success of missing data methods and ultimate statistical analyses (Saunders et al., 2006). Overall, there is no consensus on the maximum amount of missing data and numerous other factors including the sample size, type of data, and patterns of missingness must inform one's approach to dealing with missing data.

Incorporating imputation methods as standard practice for handling missing data in bioarchaeology will contribute to the advancement of the field. Numerous scholars have drawn attention to the dearth of advanced statistical analyses in bioarchaeology (Agarwal, 2016; Konigsberg & Frankenberg, 2013; Zuckerman et al., 2016). Zuckerman et al. (2016) argue that paleopathology has been slow to adopt advances from other fields, instead relying on less rigorous methods without critical reflection. These shortcomings have hindered our ability to advance our understanding of human health throughout history in a way that is meaningful to modern populations. While recent years have seen a surge in more advanced analytical methods such as hazards models, survival analysis, and principal components analysis, much research – particularly paleopathology and trauma analysis – still depends on univariate statistical analyses. While summary statistics have their place, reliance on them represents an impediment to the advancement of paleopathology. Analyses such as the *t*‐test or chi‐square have strict statistical assumptions about the data such as normal distribution or equal variances; inappropriate use of such tests can result in erroneous results and thus unfounded inferences regarding health in the past. As paleopathology data rarely adhere to these assumptions, often data must be aggregated or binned in ways that obscure vitally important patterns. More sophisticated analyses allow data to be explored in its complexity rather than by arbitrary bins, contributing to a more nuanced understanding of human health throughout history.

Another serious drawback of less advanced methods is their failure to account for the concerns raised by Wood et al. (1992) in the osteological paradox. Because of selective mortality, straightforward percentages or counts of pathology lesions from a skeletal sample will overestimate disease prevalence in the living population. The authors explain how aggregating data for more simple analyses prevents us from accounting for variation in individual frailty, obscuring not only important variation in disease experience, but also the potential presence of subpopulations.

While imputation is a vital new step forward for handling missing data in bioarchaeology, one aspect that should be considered is the use of imputed values to draw conclusions about the lived experiences of past people. A great strength of bioarchaeology is the multiscalar ability to move seamlessly from populations to individuals. Consider, for example, a bioarcheological project that incorporates biological distance and stable isotopes to examine population affinity, migration, and diet. Multidimensional scaling (MDS) plots and carbon–nitrogen graphs show population groupings and diets of the sample. Typically, the researcher would discuss the life histories of aberrant individuals that do not follow the patterns of the rest of the group, such as individuals with unusual diets or who migrated from far away. If, however, missing variables were imputed – which is already standard practice in biodistance – the locations of individual points on the plots would be based on imputed, rather than actual values which may, or may not, represent the true values. Kenyhercz and Passalacqua (2016) recommend that when selecting an imputation technique for this type of individual‐level analysis, the objective is to choose the most accurate method. However, the goal of imputation is not to recover the exact values missing from the dataset, but to “preserve important characteristics of the data set as a whole” (Graham, 2009, p. 559). Imputed data are intended to examine overall patterns in the data, not the life experiences of single individuals. How do we reconcile these two disparate goals? Further research is needed to ensure that as bioarchaeology continues to grow and adopt methods from other fields, we apply those methods appropriately.

This study has several limitations. First, according to Rubin's Rules (Rubin, 1987), statistical analyses are to be performed on each of the *m* datasets, and the final parameters of interest (e.g., *p*‐values, confidence intervals, etc.) pooled at the very end using the equations designed by Rubin. The approach used in this paper, however, pools the multiply imputed datasets and assesses success at the end – a violation of Rubin's Rules.^2^ Failing to adhere to Rubin's Rules can result in over‐ or underestimated parameters such as *SE*s, confidence intervals, and *p*‐values. Second, the success of imputation and deletion methods will depend not only on the percent of missing data, but also on the sample size. The samples used here (ordinal *n* = 287; continuous *n* = 369) are large compared to most paleopathology datasets. Additional research is needed to compare the results found here with those from smaller samples. Third, this study tests the success of missing data methods on ordinal and continuous data separately, however many bioarchaeologists collect mixed data types and analyze them together. Future research is needed to identify which methods are successful at imputing mixed data including continuous, ordinal, and binary values. Finally, this paper investigates the best methods for imputing missing data, but ultimately bioarchaeologists are interested in how those imputed values can be used to answer questions about human experiences in the past. Further research is needed to examine how imputed values perform in the types of statistical tests we use most often such as survival analysis, principle components analysis, ANOVAs, or even *t*‐tests and chi‐square tests to assess how imputed data affect our results and the conclusions we draw about past individuals and populations.

## 5 CONCLUSION

The primary aim of this paper is to provide background on missing data methods, highlighting how imputation can be used to manage missing data in bioarchaeology and paleopathology. There are a number of approaches for handling missing data, including deleting data or imputing missing values. While each technique has advantages and disadvantages, imputation methods are recommended over deletion. Other fields in the natural and social sciences commonly use imputation. However, bioarchaeological research (apart from biodistance analyses) seldom uses imputation to handle missing data. This paper tests the ability of seven methods to yield effective parameter estimates when handling missing ordinal and continuous bioarchaeological data. Results demonstrate that no single method performs best in all circumstances, suggesting there is no “one‐size‐fits‐all” solution to missing data problems. Listwise deletion is not recommended as it performed the worst for both ordinal and continuous data, introducing the most error into the dataset. While pairwise deletion preserved the characteristics of the original dataset, it is not recommended due to the loss of data. Ultimately, the best methods for handling missing data are the more sophisticated methods: stochastic regression, PMM, random forest, and EM. Overall, stochastic regression imputation consistently performed well across both ordinal and continuous data when assessed with either percent error or NRMSE.

Future studies should test the success of imputation on mixed continuous and ordinal data as well as how well imputation works with very small sample sizes. We hope these findings encourage the use of more advanced methods to manage missing data in bioarchaeology. With greater understanding of the limitations and structure of our data, bioarchaeologists can explore sources of bias more effectively and implement statistically rigorous analyses.

## AUTHOR CONTRIBUTIONS

**Amanda Wissler:** Conceptualization (lead); data curation (lead); formal analysis (lead); funding acquisition (lead); investigation (lead); methodology (lead); project administration (lead); writing – original draft (lead); writing – review and editing (equal). **Kelly E. Blevins:** Methodology (supporting); project administration (supporting); visualization (supporting); writing – original draft (supporting); writing – review and editing (equal). **Jane E. Buikstra:** Project administration (supporting); supervision (supporting); writing – original draft (supporting); writing – review and editing (equal).

## CONFLICT OF INTEREST

The authors declare no conflicts of interest.

## Supporting information


**Table S1** Results ‐ NRMSE ‐ Ordinal Data ‐ 5%, 10%, 20%, 30%, and 40% Missingness.

**Table S2** Results ‐ Percent Error ‐ Ordinal Data ‐ 5%, 10%, 20%, 30%, and 40% Missingness.

**Table S3** Results ‐ NRMSE ‐ Ordinal Data ‐ MCAR, MAR, and MNAR Missingness.

**Table S4** Results ‐ Percent Error ‐ Ordinal Data ‐ MCAR, MAR, and MNAR.

**Table S5** Results ‐ NRMSE ‐ Continuous Data ‐ 5%, 10%, 20%, 30%, and 40% Missingness.

**Table S6** Results ‐ Percent Error ‐ Continuous Data ‐ 5%, 10%, 20%, 30%, and 40% Missingness.

**Table S7** Results ‐ NRMSE ‐ Continuous Data ‐ MCAR, MAR, and MNAR Missingness.

**Table S8** Results ‐ Percent Error ‐ Continuous Data ‐ MCAR, MAR, and MNAR Missingness.

**Table S9** Overall Results ‐ Average of Averages.

## Data Availability

The R code created for this study is openly available via the first author's GitHub Repository (Wissler, 2022). The raw paleopathology data that support the findings of this study are available from the Cleveland Museum of Natural History with permission from the first author. Data will be available by contacting the Collections Manager of Physical Anthropology.

## Endnotes

^1^
Fraction of missing information (FMI) is “a measure of uncertainty about the values we would impute for missing elements” (Wagner, 2010; p. 224). It quantifies the amount of variance between imputed datasets and is affected by both the quality and quantity of available data. See Wagner (2010) for full discussion of FMI and the mathematical definition.

^2^
Recent research has shown that Rubin's Rules can create biased variance estimates depending on the imputation method used and therefore may not always be the best approach for pooling parameter estimates. See Bartlett (2021) and Seamen et al. (2014) for further discussion.
